# Rational and combinatorial approaches to engineering styrene production by *Saccharomyces cerevisiae*

**DOI:** 10.1186/s12934-014-0123-2

**Published:** 2014-08-21

**Authors:** Rebekah McKenna, Brian Thompson, Shawn Pugh, David R Nielsen

**Affiliations:** Chemical Engineering, School for Engineering of Matter, Transport, and Energy, Arizona State University, PO Box 876106, Tempe, AZ 85287-6106 USA

**Keywords:** Styrene, Yeast, Phenylalanine, Aromatics

## Abstract

**Background:**

Styrene is an important building-block petrochemical and monomer used to produce numerous plastics. Whereas styrene bioproduction by *Escherichia coli* was previously reported, the long-term potential of this approach will ultimately rely on the use of hosts with improved industrial phenotypes, such as the yeast *Saccharomyces cerevisiae*.

**Results:**

Classical metabolic evolution was first applied to isolate a mutant capable of phenylalanine over-production to 357 mg/L. Transcription analysis revealed up-regulation of several phenylalanine biosynthesis pathway genes including *ARO3*, encoding the bottleneck enzyme DAHP synthase. To catalyze the first pathway step, phenylalanine ammonia lyase encoded by *PAL2* from *A. thaliana* was constitutively expressed from a high copy plasmid. The final pathway step, phenylacrylate decarboxylase, was catalyzed by the native *FDC1*. Expression of *FDC1* was naturally induced by *trans*-cinnamate, the pathway intermediate and its substrate, at levels sufficient for ensuring flux through the pathway. Deletion of *ARO10* to eliminate the competing Ehrlich pathway and expression of a feedback-resistant DAHP synthase encoded by *ARO4*^*K229L*^ preserved and promoted the endogenous availability precursor phenylalanine, leading to improved pathway flux and styrene production. These systematic improvements allowed styrene titers to ultimately reach 29 mg/L at a glucose yield of 1.44 mg/g, a 60% improvement over the initial strain.

**Conclusions:**

The potential of *S. cerevisiae* as a host for renewable styrene production has been demonstrated. Significant strain improvements, however, will ultimately be needed to achieve economical production levels.

**Electronic supplementary material:**

The online version of this article (doi:10.1186/s12934-014-0123-2) contains supplementary material, which is available to authorized users.

## Background

Similar to most other monomers used in conventional plastics production, at present, styrene is derived exclusively from non-renewable feedstocks. More specifically, current styrene production predominantly involves the energy-intensive chemocatalytic dehydrogenation of petroleum-derived ethylbenzene [1,2]. With the global annual demand of styrene expected to surpass 41 million tons by 2020 [3], representing a > $28 billion U.S. market [4], net energy requirements associated with just this single conversion will amount to over 200 trillion BTU of steam each year [5]. Accordingly, concerns over depleting feedstock availability and deleterious environmental impacts, continue to drive interest in developing ‘green’ processes for producing biorenewable replacements to conventional petrochemicals, including monomers such as styrene.

**Figure 1:**
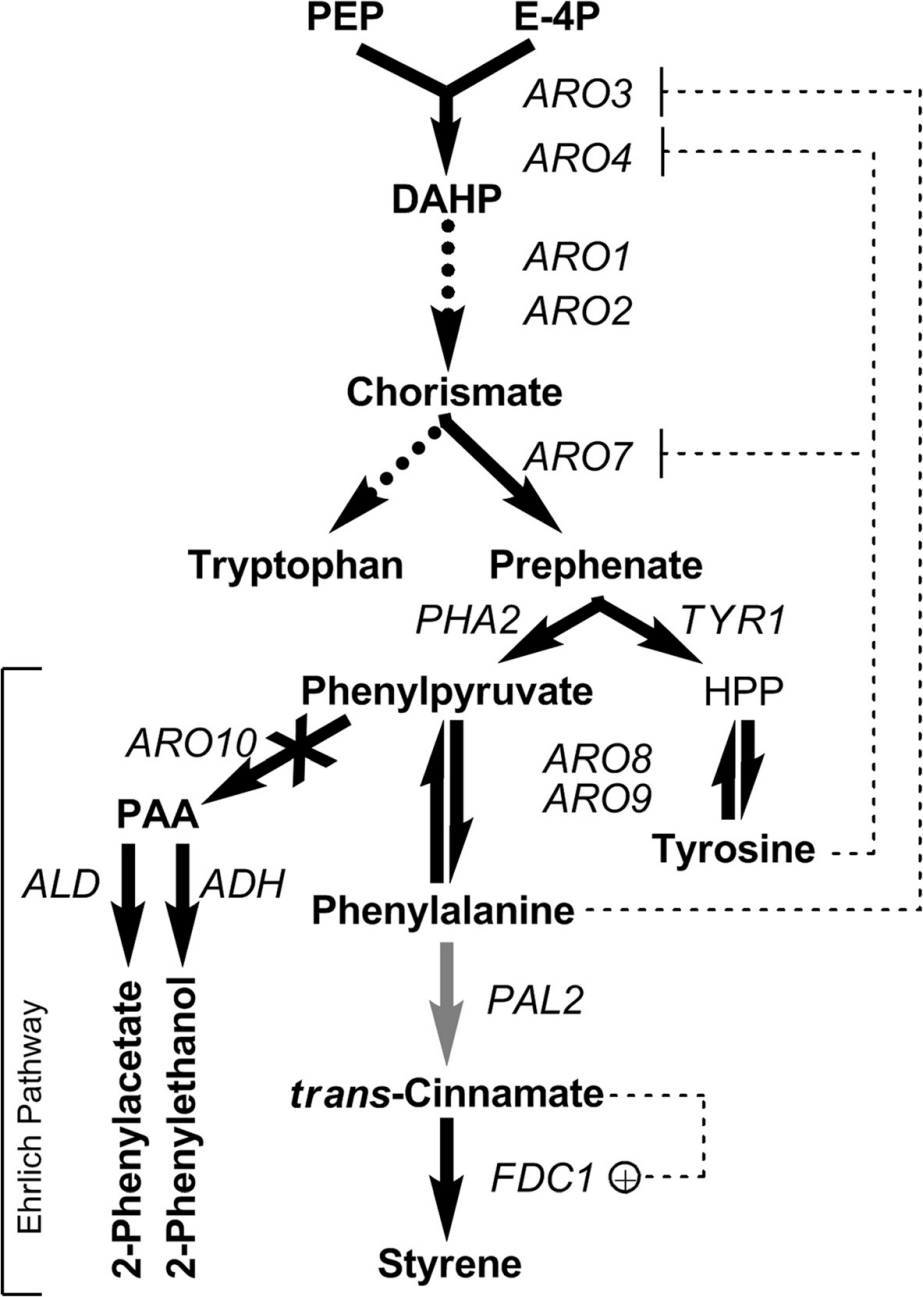
**Styrene biosynthesis by engineered S. cerevisiae.** Dashed arrows signify multiple steps are involved but not illustrated. Black arrows represent enzymes steps native to S. cerevisiae whereas gray arrows are heterologous; dotted arrows represent multiple enzymatic steps; feedback repression is shown using thin dotted lines with flat heads whereas transcriptional activation is shown using thin dotted lines with a round head and ‘+’; disruption of a gene or regulatory mechanism is signified by ‘X’. Abbreviations: phosphoenolpyruvate (PEP), erythrose-4-phosphate (E-4P), 3-deoxy-D-arabino-heptulosonate-7-phosphate (DAHP), hydroxyphenylpyruvate (HPP), 2-phenylacetaldehyde (PAA).

Advances in metabolic and pathway engineering continue to expand the range of conventional monomer compounds that can be synthesized from renewable biomass feedstocks [6-9]. Along these lines, the engineering of a novel and non-natural pathway for styrene biosynthesis from biomass-derived glucose was recently reported using the bacterium *Escherichia coli* as host [10]. Said pathway, which is illustrated in Figure 1, utilizes endogenous phenylalanine as its immediate precursor. Phenylalanine is first deaminated to *trans*-cinnamate by phenylalanine ammonia lyase (PAL), encoded by *PAL2* from *Arabidopsis thaliana*. Next, *trans*-cinnamate is decarboxylated to styrene via phenylacrylate decarboxylase, encoded by *FDC1* from *Saccharomyces cerevisiae* [10,11]. Co-expressing *PAL2* and *FDC1* in a previously-engineered phenylalanine over-producing *E. coli* background resulted in styrene titers as high as ~260 mg/L (2.5 mM) in glucose minimal media after 29 h, representing a glucose yield of ~0.07 g/g (0.12 mol/mol; 25% of theoretical) [10]. As a commodity chemical, however, said production metrics must be improved for biologically derived styrene to emerge as a viable alternative to its conventional counterpart [12].

The engineering of more robust hosts for renewable chemical production is an important aim in industrial biotechnology [13] and, relative to *E. coli*, yeast biosynthetic platforms often afford several inherent attributes of importance to robust and large–scale renewable chemicals production. Said phenotypes most significantly include faster growth rates, elevated solvent tolerance, and the ability to withstand low temperatures and pH [14-18]. Amongst yeast, *S. cerevisiae* is a particularly attractive host for metabolic engineering efforts owing to its well characterized genetics, physiology, and metabolism, as well as due to the availability of diverse genetic toolkits for its engineering [19]. Past studies have demonstrated that *S. cerevisiae* is a suitable host for the renewable production of useful aromatic compounds, including protocatechuate, catechol, vanillin, naringenin, and 2-phenylethanol [20-23], production of styrene, however, has not yet been reported.

The objective of the present study was to demonstrate that styrene biosynthesis from glucose could be systematically engineered in *S. cerevisiae*. In addition to the aforementioned phenotypic advantages afforded by the use of *S. cerevisiae*, this aim was further motivated by other relevant factors. For instance, it was hypothesized that improved function of styrene pathway enzymes might be realized in *S. cerevisiae* since: *i) PAL2* is of eukaryotic origin, and *ii) FDC1* is native to *S. cerevisiae.* Furthermore, contingent upon the native regulation of *FDC1* expression (as investigated in this study), it is plausible that a functional styrene pathway could be constructed by expressing a single heterologous enzyme (i.e., *PAL2*), thereby minimizing the effects of metabolic burden. Meanwhile, as *S. cerevisiae* lacks a natural transporter for phenylalanine efflux [24], it was hypothesized that increased intracellular retention of phenylalanine might enhance its availability to the engineered pathway. These unique features position *S. cerevisiae* as particularly promising host for renewable styrene production. In this study, classical anti-metabolite selection was first applied to evolve a *S. cerevisiae* strain capable of over-producing phenylalanine, the styrene pathway precursor. Rational genetic engineering approaches were used to construct the non-natural styrene pathway and further boost precursor availability. While not before applied for styrene bioproduction, this basic approach has been proven effective for engineering bacterial producers of other aromatic chemicals [25-28].

## Results and discussion

### Evolving phenylalanine over-production by S. cerevisiae

**Figure 2:**
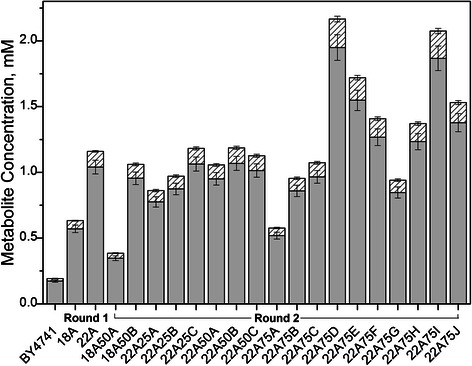
**Evolution of phenylalanine overproducing mutants of** ***S. cerevisiae*** **.** Mutants were evolved through the use of EMS mutagenesis and high-throughput selection on solid agar plates using *m*-fluoro-DL-phenylalanine as anti-metabolite. 2-Phenylethanol (gray) and 2-phenylacetate (hashed) production by isolated mutants was determined after 48 h of growth in SD media by measuring the concentration of 2-phenylethanol and 2-phenylacetate in the supernatant. Error bars reported at one standard deviation from triplicate experiments.

As phenylalanine serves as the immediate endogenous precursor to the styrene pathway, its over-production by *S. cerevisiae* is an essential pre-requisite to styrene biosynthesis. Thus, to develop a phenylalanine over-production phenotype in *S. cerevisiae*, a classic antimetabolite selection strategy was first employed [29]. In this case, *m*-fluoro-DL-phenylalanine was chosen to provide the necessary selection pressure whereas exposure to the chemomutagen EMS increased mutation rates and frequency [30]. In the first round of selection, a total of only two mutants were isolated when using either 18 mg/L (strain 18A) or 22 mg/L (strain 22A) *m*-fluoro-DL-phenylalanine. As seen in Figure 2, said mutants were subsequently characterized in shake flask cultures. Since phenylalanine is not exported from *S. cerevisiae*, the established practice of correlating enhanced flux through the pathway with net extracellular accumulation of 2-phenylethanol and 2-phenylacetate (both of which are naturally and readily produced as degradation products of phenylpyruvate, the precursor to phenylalanine; Figure 1) was employed [24]. In *S. cerevisiae*, it has previously been shown that all available phenylalanine is efficiently shuttled through one or both of these pathways [31], with the relative distribution of products being dictated by the cell’s redox state (specifically, the relative intracellular ratio of NAD + to NADH) [32]. For example, in glucose grown cultures with limited aeration (as would be expected in the sealed shake flasks used in this study), 9:1 mixtures of 2-phenylethanol:2-phenylacetate are typically observed [32]. Throughout this study, the mixture of products obtained was similarly consistent (an average of 89% 2-phenylethanol; Figure 2). Relative to BY4741 (the parent strain and control), strains 18A and 22A showed 3.3- and 6.4-fold improvements in net production of 2-phenylethanol and 2-phenylacetate (0.63 ± 0.05 and 1.13 ± 0.10 mM), respectively.

To further deregulate and enhance phenylalanine biosynthesis, a second round of mutagenesis and selection was performed, in this case using the isolated mutants 18A and 22A as parents. The selection pressure was accordingly elevated by increasing the content of *m*-fluoro-DL-phenylalanine in SD agar plates to 25 mg/L, 50 mg/L, or 75 mg/L. In contrast to the first round, in this case numerous colonies (i.e., > 50) were obtained at all three selection pressures. Thus, to screen just for the best performers, only the fastest growing mutants were selected (i.e., those whose colony forming units were largest after overnight incubation). A total of 18 additional mutants were chosen and subsequently characterized, as above. As seen in Figure 2, the top performing mutant, 22A75D, produced a combined total of about 2.17 ± 0.22 mM 2-phenylethanol and 2-phenylacetate in 48 h, representing ~3.3- and ~21-fold improvements over 22A and BY4741, respectively.

### Investigating the evolved phenotypes

A series of characterizations were next performed on strains 22A and 22A75D (with BY4741 as control) to begin to understand the underlying factors responsible for imparting the evolved phenotypes. The native aromatic amino acid biosynthesis pathways of *S. cerevisiae* are shown in Figure 1, where it can be seen that two known control points are principally responsible regulating metabolite flux. The first occurs at DAHP synthase (for which *S. cerevisiae* possesses two isoenzymes), which is allosterically feedback inhibited by either phenylalanine (ARO3) or tyrosine (ARO4) [33-35]. The second, meanwhile, occurs at chorismate mutase (ARO7), which converts chorismate to prephenate, the precursor to both phenylalanine and tyrosine. Transcription of ARO7 is repressed in the presence of as little as 0.5 mM tyrosine but remains, however, insensitive to phenylalanine [33,36,37]. Here, overcoming feedback repression of ARO3 thus constitutes a key priority. However, whereas relief from tyrosine repression of ARO4 has been reported to result from a single mutation (K229L) [24], a phenylalanine feedback resistant mutant of ARO3 remains unreported to date.

**Figure 3:**
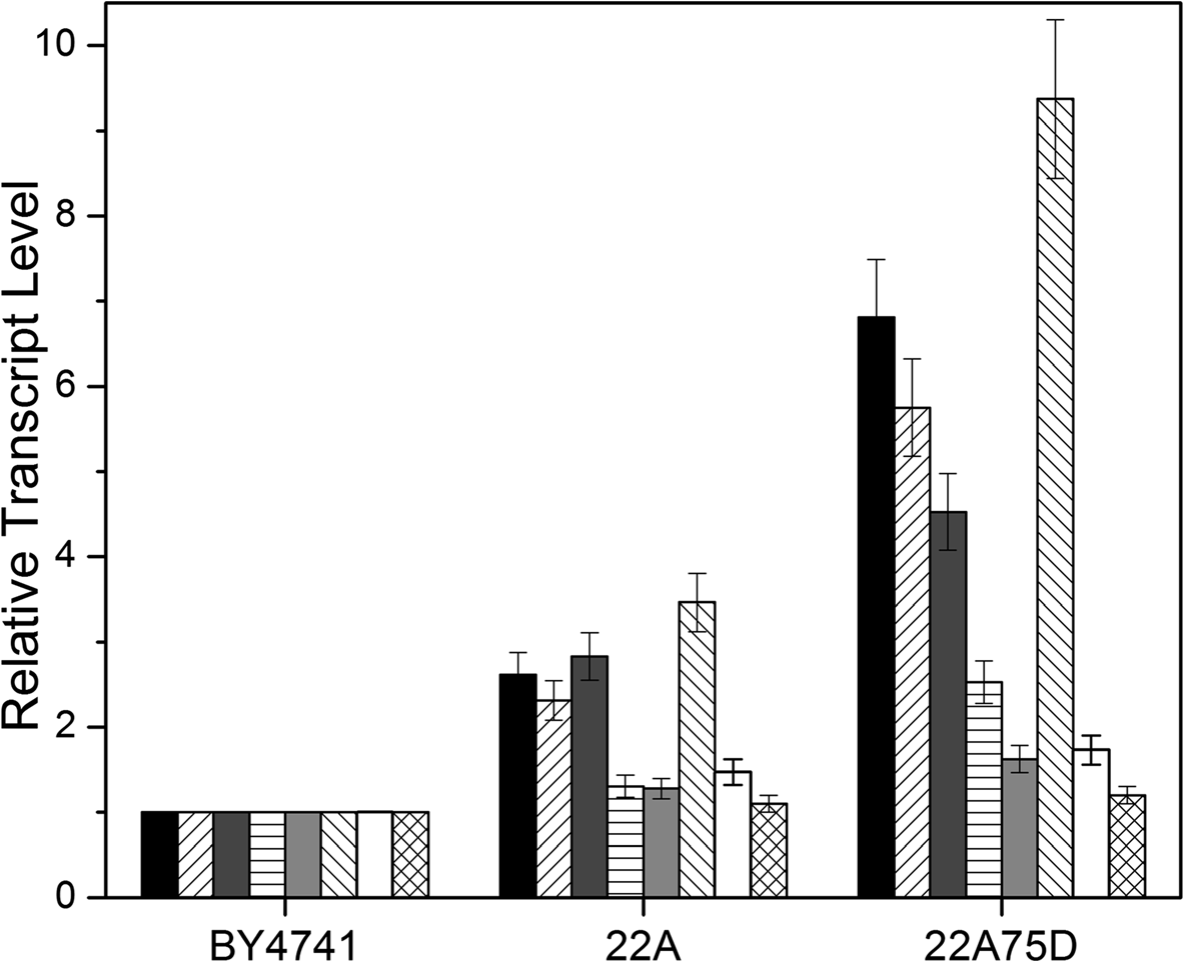
**Transcriptional analysis of top phenylalanine overproducing** ***S. cerevisiae*** **mutants.** Relative transcript levels of the top first (22A) and second (22A75D) round evolved yeast mutants, normalized to the parent (BY4741). Measured genes included *ARO1* (black), *ARO2* (right diagonal), *ARO3* (dark gray), *ARO4* (horizontal), *ARO7* (light gray), *ARO8* (left diagonal), *ARO9* (no fill), and *PHA2* (hashed). Error bars reported at one standard deviation from triplicate experiments.

Sequences of several key genes in the phenylalanine biosynthesis pathway (*ARO3*, *ARO4*, *ARO7*, *ARO8,* and *PHA2*) were first determined for all three strains (including coding regions as well as 500 bp upstream of each start codon). Interestingly, however, mutations were not observed in the sequence of any investigated gene, including with respect to both its coding and upstream non-coding regions. Transcription levels of all genes in the phenylalanine biosynthesis pathway (namely *ARO1*, *ARO2*, *ARO3*, *ARO4*, *ARO7*, *ARO8*, *ARO9*, and *PHA2*; see Figure 1) were next examined in the mutants 22A and 22A75D and quantified relative to that of the wild-type control (BY4741). The results are compared in Figure 3, wherein it can be seen that, in strain 22A75D, up-regulation of *ARO8* was found to be most significant (a 9.3-fold increase), followed by *ARO1* (6.8-fold), *ARO2* (5.8-fold), and *ARO3* (4.5-fold). Note that similar but less significant differences were also observed in strain 22A. Furthermore, only modest increases in *ARO4* and *ARO7* expression were observed in 22A75D (about 2.7- and 1.8-fold, respectively), with no significant changes occurring in 22A for either gene.

The collective findings point to the prospects of several interesting mechanisms in the mutant strains. For example, with no change to its sequence, the evolved phenotype clearly did not arise as a result of relieving allosteric inhibition at the known bottleneck enzyme, ARO3. However, as a significant increase in its expression was observed in both mutants, this could suggest that up-regulation of wild type *ARO3* occurred as an alternative strategy. That is, despite the fact that the wild type enzyme possesses lower specific activity in the presence of phenylalanine, with more copies net DAHP synthase activity may have been sufficiently high so as to effectively overcome the flux bottleneck. Simultaneous up-regulation of *ARO1* and *ARO2* (the two subsequent steps in the pathway), meanwhile, may have aided in this process by ensuring that produced DAHP was then promptly assimilated further along the pathway, thereby maintaining a maximum driving force. Up-regulated expression of *ARO1* has been successfully employed in yeast as a rational approach for enhancing the biosynthesis of *cis,cis-*muconic acid – a product derived from the shikimate pathway intermediate 3-hydroshikimate [38]. Meanwhile, up-regulation of *ARO8* was evolved perhaps as a mechanism to compete with the native activity of *ARO10*, ensuring that metabolite flux was efficiently routed towards phenylalanine biosynthesis rather than through the degradative Ehrlich pathway [32,39]. This prospect is further supported by the fact that, in contrast to *ARO8*, no appreciable change was observed with respect to the expression of *ARO9*, which functions primarily in the reverse direction (i.e., for phenylalanine assimilation from the culture medium) [40].

With no change to either the coding or upstream non-coding regions for any of the four up-regulated genes (*ARO1*, *ARO2*, *ARO3*, and *ARO8*) another factor must be responsible for this observed result. Increased copy number through genomic amplifications is one possibility, however, a more efficient mechanism may have involved the mutation of one or more transcription factors controlling their expression. *GCN4* encodes one such major transcription factor [41], however, further sequencing revealed no changes there either. To identify other prospective transcription factors involved, the promoter regions of the four up-regulated genes were further investigated by aligning the 1000 bp sequences prior to each start codon. Whereas a possible consensus sequence of 5’-AACATC-3’ was located at positions −292, −307, −289, and −290 for *ARO1*, *ARO2*, *ARO3*, and *ARO8*, respectively, said sequence does not match binding site of any known transcription factor. Among all known transcriptional regulators, eleven are shared between the four up-regulated genes [42] (note, an annotated list is provided in Additional file 1: Table S3). The ability to determine which if any of these regulators are responsible for the evolved changes will only be possible through the collective analysis of their gene sequences, or better, to ensure full elucidation of all changes in the mutants, through whole-genome sequencing. However, given that the achievable titers are still quite modest (a total of only 2.17 ± 0.22 mM 2-phenylethanol and 2-phenylacetate), such an undertaking was deemed as unwarranted at this time. For now, and for the purpose of this study, these efforts were successful in developing a host strain to serve as a test platform for engineering styrene biosynthesis from glucose.

### Investigating native FDC1 activity and factors influencing its expression

Although it had previously been shown that, when cultured in the presence of exogenous *trans*-cinnamate, *S. cerevisiae* is capable of catalyzing its decarboxylation to styrene [11], several factors related to the native function and expression of *FDC1* remained initially unclear and deserving of further investigation. Most importantly, it was wholly unknown as to if, when, and how the native expression of *FDC1* would be induced in the context of the styrene pathway and under the culture conditions of interest. BY4741 was initially cultured in SD minimal media supplemented with potential inducers of interest. In addition to *trans*-cinnamate and phenylalanine (the pathway intermediate and precursor, respectively), *p-*coumarate and ferulate were also screened as positive controls (note, both are structural homologs of *trans*-cinnamate and known inducers of *trans*-cinnamate decarboxylase activity [11]). As seen in Table 1, *in vitro trans-*cinnamate decarboxylase activity was detected in the lysates of cells cultured in the presence of each of *trans-*cinnamate, *p-*coumarate, and ferulate (with the former serving as the strongest inducer), but not with phenylalanine or in the control. With respect to the styrene pathway, this implies that native *FDC1* expression will be wholly contingent upon the heterologous expression of *PAL2* to provide *trans-*cinnamate as inducer (a realization that could be of benefit with respect to minimizing overall metabolic burden).

**Table 1:** **Assaying the in vitro decarboxylase activity of FDC1 against a pool of structurally-related, phenylacrylic acid substrates**

**Compound**	**Induced activity**	**mU mg** ^**−1**^ **total protein**
*trans*-cinnamate	+	0.46 ± 0.02
*p*-coumarate	+	0.39 ± 0.02
ferulate	+	0.21 ± 0.03
phenylalanine	-	N.D.
none (control)	-	N.D.

Positive, ‘+’; Negative, ‘-‘; Not Detected, ‘N.D.’.

### Evaluating the styrene pathway via the exogenous addition of phenylalanine

**Figure 4:**
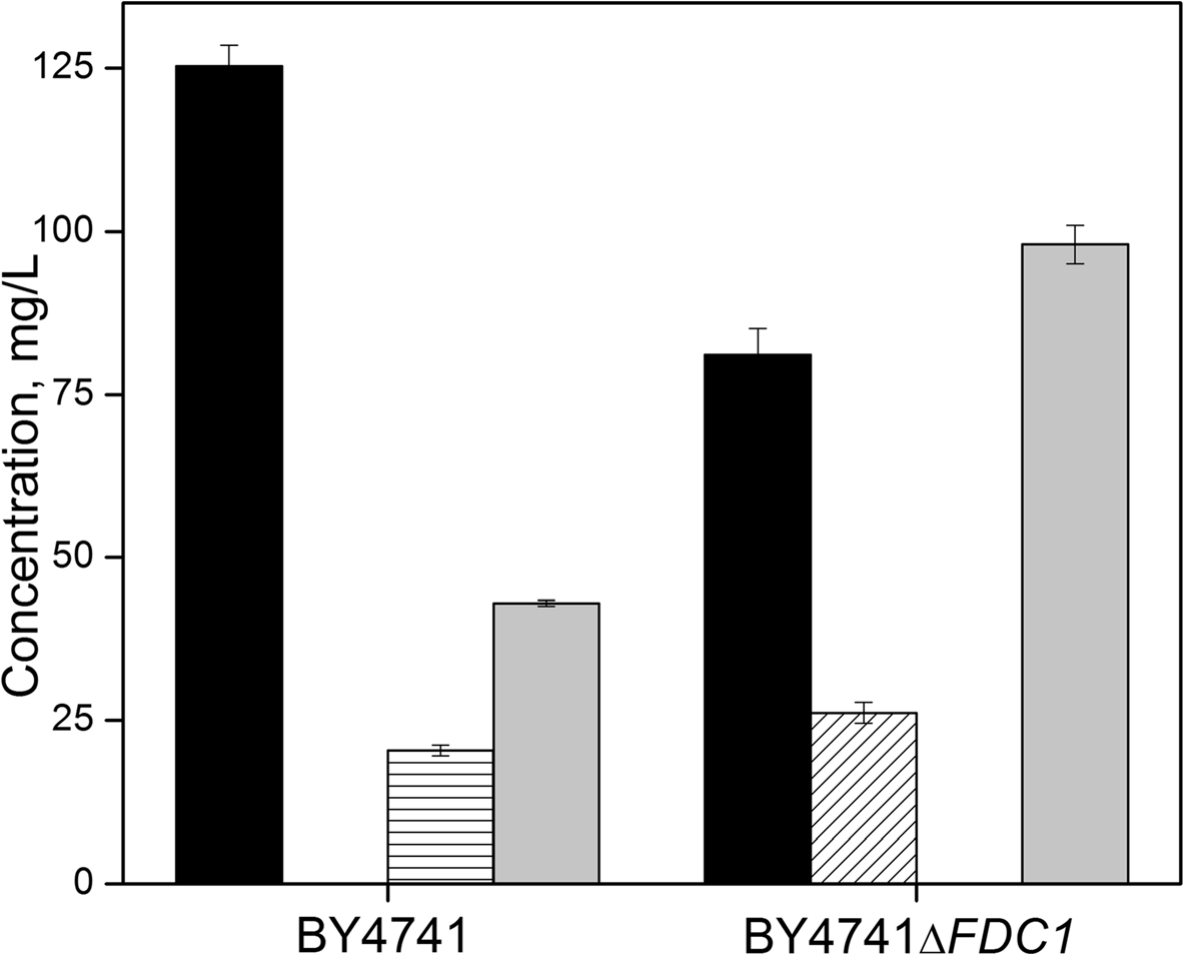
**Assessing the trans-membrane export of** ***trans*** **-cinnamate.** Depletion of exogenous phenylalanine (black; initially 200 mg/L) by growing cultures of wild-type *S. cerevisiae* BY4741-PAL and BY4741*ΔFDC1*-PAL and the resultant production of *trans*-cinnamate (diagonal), styrene (horizontal), and 2-phenylethanol (gray) after 24 h. Error bars reported at one standard deviation from triplicate experiments.

Preliminary studies were next performed to begin probing the functionality of the styrene pathway in wild type *S. cerevisiae*. Strains BY4741-PAL and BY4741*ΔFDC1*-PAL were first cultured in SD-Leu minimal media supplemented with 200 mg/L (1.21 mM) phenylalanine while monitoring the extracellular accumulation of *trans*-cinnamate, styrene, and 2-phenylethanol, 2-phenylacetate, and concomitant depletion of phenylalanine. As seen in Figure 4, while only 37% of supplied the phenylalanine was consumed by BY4741-PAL after 24 h, styrene and 2-phenylethanol constituted the major end-products, reaching titers of up to 20 ± 1 and 43 ± 1 mg/L (0.19 ± 0.01 and 0.35 ± 0.01 mM), respectively, with *trans*-cinnamate being undetected. In contrast, 2-phenylethanol was produced to a final titer of 98 ± 3 mg/L (0.80 ± 0.02 mM) by BY4741*ΔFDC1*-PAL with styrene being undetected throughout. In addition, in this case the extracellular accumulation of *trans*-cinnamate was also observed, reaching final concentration of 26 ± 3 mg/L (0.18 ± 0.02 mM) by 24 h. Only trace levels of 2-phenylacetate were observed throughout.

These results demonstrate several key points. First, styrene can be produced from phenylalanine by *S. cerevisiae* via the heterologous expression of *PAL2* and native expression of *FDC1*. Second, both styrene and *trans*-cinnamate are naturally excreted from *S. cerevisiae*, at least to a certain degree. Third, since *trans*-cinnamate did not accumulate in BY4741-PAL cultures, this implies that the net activity afforded by native *FDC1* expression was sufficiently high so as to preclude the creation of a flux bottleneck at the final pathway step (at least with respect to the specific *PAL2* expression level examined). And, lastly, that under the conditions examined, synthesis of byproduct 2-phenylethanol significantly competes with the styrene pathway for precursor availability, even when *PAL2* is constitutively expressed on a high copy number plasmid.

### Styrene production from glucose

**Figure 5:**
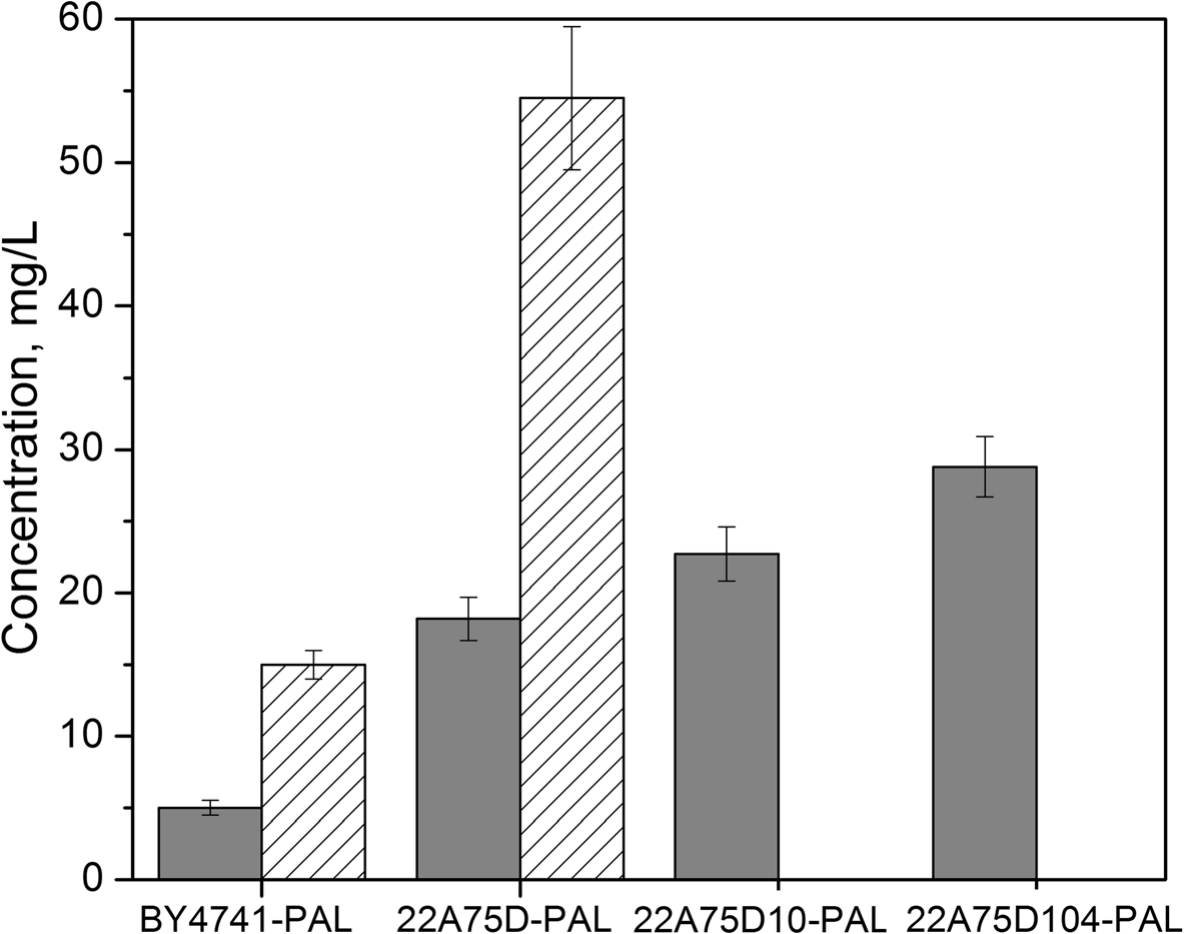
**Styrene biosynthesis from glucose by engineered** ***S. cerevisiae*** **strains.** Styrene (gray) and 2-phenylethanol (lined) production by strains BY4741-PAL, 22A75D-PAL, 22A75D10-PAL, and 22A75D104-PAL after 48 h in SD-Leu minimal media in sealed shake flask cultures. Error bars reported at one standard deviation from triplicate experiments.

Based on the above findings, a series of strains were next constructed and evaluated with respect to their styrene production potential from glucose, the results of which are compared in Figure 5. Although only as much as ~5 mg/L (0.05 mM) styrene was detected in BY4741-PAL cultures, 22A75D-PAL accumulated up to 18 ± 2 mg/L (0.17 ± 0.02 mM) styrene in 48 h. In the latter culture, however, more significant accumulation of byproduct 2-phenylethanol was also observed, reaching up to 54 ± 5 mg/L (0.44 ± 0.04 mM; again, 2-phenylacetate did not accumulate above trace levels). As this again suggested that the native Ehrlich pathway was competitively inhibiting the styrene pathway with respect to precursor availability, the effect of deleting *ARO10* – which converts phenylpyruvate to phenylacetaldehyde [32,43] – on styrene production was explored. As seen in Figure 5, deletion of *ARO10* to preserve phenylpyruvate availability improved styrene production by 22A75D10-PAL by ~28%, reaching up to 23 ± 2 mg/L (0.22 ± 0.02 mM). Lastly, to further improve styrene production, a feedback resistant mutant of *ARO4* – namely *ARO4*^*K229L*^ – was introduced into 22A75D10-PAL. Although *ARO4* encodes a tyrosine-sensitive DAHP synthase, in previous works *ARO4*^*K229L*^ over-expression in *S. cerevisiae* was shown to increase flux through the shikimic acid pathway by as much as 4.5-fold [16,24]. Here, expressing *ARO4*^*K229L*^, 22A75D104-PAL displayed an additional 25% increase in styrene titer, reaching up to 29 ± 2 mg/L (0.28 ± 0.02 mM) at a glucose yield of about 1.44 ± 0.11 mg/g (0.0025 ± 0.0002 mol/mol; or just 0.6% of theoretical). Meanwhile, *trans*-cinnamate was not detected in the culture media of any styrene producing strain at any time (data not shown).

As a volatile product, it was also noted that styrene significantly accumulated within the headspace of each sealed flask. We have previously confirmed that said vapor–liquid partitioning behaves according to Henry’s Law, with a dimensionless Henry’s Law coefficient of 0.113 at 32°C [10]. For the conditions examined here, this meant that an additional ~45% styrene was produced and accumulated in each case. With this in mind, the maximum volumetric styrene production achieved here would be more accurately represented as 42 ± 3 mg/L (0.40 ± 0.03 mM) with a glucose yield of 2.09 ± 0.16 mg/g (0.0036 ± 0.0003 mol/mol; 0.9% of theoretical). The volatile nature of styrene may also prove useful as a product recovery strategy in the future [44].

Even at this adjusted output, however, achievable styrene production remains only 18% of the net production of 2-phenylethanol and 2-phenylacetate demonstrated by 22A75D (2.17 ± 0.22 mM) and is 65% of the net production of styrene and 2-phenylethanol by 22A75D-PAL (0.61 ± 0.02 mM). This suggests that multiple limiting factors may have arisen during strain construction. Although it was not anticipated to be problematic at such low aqueous titers, to ensure that end-product inhibition was not the central productivity-limiting factor, a cursory evaluation of styrene toxicity was performed. *S. cerevisiae* growth and viability was found to be only minimally disrupted in the presence of styrene at up to at least 200 mg/L (1.92 mM; data not shown), suggesting that styrene toxicity was not a critical barrier at this point. Over-expression of *PAL2* appears to have had the greatest negative impact, reducing net aromatic production by 72%. This was likely due to the metabolic burden imposed by its expression from a high-copy plasmid. While decreasing *PAL2* expression will likely reduce burden, as *trans*-cinnamate was never detected in styrene producing cultures, this already points to the fact that PAL2 activity was rate-limiting in the styrene pathway. Thus, future and careful optimization of *PAL2* expression and/or the identification of other PAL homologs displaying greater inherent activity in *S. cerevisiae* will be key to achieving further improvements in styrene production.

Lastly, although seemingly low, it should be appreciated that level of styrene production demonstrated here agrees well with that of prior reports by others whom have engineered *S. cerevisiae* to produce aromatic chemicals (for which Curran *et al.* previously provided a comprehensive examination). Notable examples include *p-*hydroxycinnamate [45], *p-*aminobenzoic acid [46], *p-*hydroxybenzoic acid [46], and vanillin [20], which have been produced to maximal titers of up to 33.3, 34.3, 89.8, and 45 mg/L, respectively, at yields of 1.7, 2.3, 6.0, and 2.3 mg/g. Meanwhile, when compared with the baseline for styrene production established using an *E. coli* platform [10], the achievable titers and yields demonstrated with *S. cerevisiae* currently lag by about 9- and 21-fold, respectively. To achieve higher styrene titers with *S. cerevisiae*, further de-regulation of metabolite flux through its phenylalanine biosynthesis pathway will ultimately be required.

## Conclusions

By coupling the classical approach of metabolic evolution with systematic strain and pathway engineering, styrene bioproduction directly from glucose by *S. cerevisiae* has been demonstrated for the first time. While providing an important demonstration of concept, future strain engineering efforts will be required to ultimately achieve economical production levels.

## Materials and methods

### Strains and media

All strains and plasmids used in this study are listed in Table 2. Custom oligonucleotide primers were synthesized by Integrated DNA Technologies (Coralville, IA) and are provided in Additional file 1: Table S1. All *S. cerevisiae* strains were purchased from Thermo Scientific (Waltham, MA). Yeast plasmids used were derived from the Gateway vector collection and purchased from AddGene (Cambridge, MA). Genomic DNA was prepared from *S. cerevisiae* whole cells using the ZR Fungal/Bacterial DNA MiniPrep (Zymo Research, Irvine, CA) according to vendor protocols. *E. coli* strain NEB10β (New England Biolabs, Ipswich, MA) was used for routine cloning and plasmid propagation, except for pDONR221 which was propagated in One Shot *ccdB* Survival 2 T1 *E. coli* (Life Technologies, Grand Island, NY). *E. coli* strains were routinely cultured at 37°C in Luria-Bertani (LB) broth supplemented with appropriate antibiotics, as necessary. Yeast strains were routinely cultured at 32°C in Yeast Extract Peptone Dextrose (YPD) medium, yeast synthetic dextrose (SD) medium, or yeast synthetic minimal (SD-Leu) medium. YPD medium was composed of 10 g/L yeast extract, 20 g/L peptone, and 20 g/L glucose. SD medium was composed of 6.7 g/L yeast nitrogen base, 20 g/L glucose, and 20 mg/L of each uracil, histidine, leucine, and methionine. SD-Leu medium was composed of 6.7 g/L yeast nitrogen base, 20 g/L glucose, 20 mg/L each uracil, histidine, and methionine.

**Table 2:** **List of strains and plasmids engineered and/or used in this study**

**Strain**	**Genotype**	**Source**
*E. coli*		
NEB10β	*araD139 ∆(ara,leu)7697 fhuA lacX74 galK16 galE15 mcrA f80d(lacZ∆M15)recA1 relA1 endA1 nupG rpsL rph spoT1∆(mrr-hsdRMS-mcrBC)*	New England Biolabs
One Shot *ccdB* Survival 2 T1	F^−^ *mcr*A Δ(*mrr*-*hsd*RMS-*mcr*BC) Φ80*lac*ZΔM15 Δ*lac*X74 *rec*A1 *ara*Δ139 Δ(*ara*-*leu*)7697*gal*U *gal*K *rps*L (Str^R^) *end*A1 *nup*G *fhu*A::*IS2*	Life Technologies
*S. cerevisiae*		
BY4741	*MATa his3Δ0 leu2Δ0 met15Δ0 ura3Δ0*	Thermo Scientific
BY4741*ΔFDC1* (YDR539W)	*MATa his3Δ0 leu2Δ0 met15Δ0 ura3Δ0 fdc1Δ*	Thermo Scientific
22A	phenylalanine overproducer evolved from BY4741	This Study
22A75D	phenylalanine overproducer evolved from 22A	This Study
BY4741-PAL	*MATa his3Δ0 leu2Δ0 met15Δ0 ura3Δ0* p425GPDPAL	This Study
BY4741*ΔFDC1*-PAL	*MATa his3Δ0 leu2Δ0 met15Δ0 ura3Δ0 fdc1Δ* p425GPDPAL	This Study
22A75D-PAL	22A75D harboring p425GPDPAL	This Study
22A75D10-PAL	22A75D *aro10Δ* harboring p425GPDPAL	This Study
22A75D104-PAL	22A75D *aro10Δ::aro4* ^*K229L*^ harboring p425GPDPAL	This Study
**Plasmids**		
pTrc99A	*P*_*trc*_, pBR322 ori, *lacI* ^*q*^ *, Amp* ^*R*^	Prather Lab, MIT
pFA6-KanMX	KanR2, pBR322 ori, *Amp* ^*R*^	AddGene
pDONR221	*att*P1-*ccdB*-*Cm* ^*R*^-*att*P2 cassette, pUC ori, *Kan* ^*R*^	Life Technologies
pDONR-PAL	*PAL2* from *A. thaliana* inserted into pDONR221	This study
p425GPD	*P*_*GPD*_, *att*R1-*ccdB*-*Cm* ^*R*^-*att*R2 cassette, pBR322 ori, *LEU2*, *Amp* ^*R*^	AddGene
p425GPDPAL	*PAL2* from *A. thaliana* inserted into p425GPD	This study
pACYCDuet-1	*P*_*T7lac*_, p15A ori, *lacI* ^*q*^ *, Cm* ^*R*^	Novagen
pACYC-ARO4^K229L^-KanMX	pACYCDuet-1 with the integration cassette *aro4* ^*K229L*^-KanMX	This Study
pUN15-PAL2	Clone U12256 containing AT3G53260 (*PAL2*) from *A. thaliana*	ABRC

### Evolution of phenylalanine over-producing mutants

Evolution of a phenylalanine over-producing phenotype in *S. cerevisiae* was achieved through random mutagenesis and high-throughput selection. The phenylalanine anti-metabolite *m*-fluoro-DL-phenylalanine provided selective pressure. *S. cerevisiae* BY4741 was first treated with ethylmethanesulphonate (EMS) according to standard protocols [47] before then being plated on minimal media supplemented with *m*-fluoro-DL-phenylalanine. In the first round of mutagenesis, selection occurred on SD media plates supplemented with either 18 or 22 mg/L *m*-fluoro-DL-phenylalanine. Note that the minimum inhibitory concentration of *m*-fluoro-DL-phenylalanine against BY4741 was initially determined as ~15 mg/L (data not shown). Mutants isolated from the first round were then subjected to a second round of mutagenesis and selection, however, in this case using 25, 50, or 75 mg/L *m*-fluoro-DL-phenylalanine. All isolated mutants were then cultured in SD media at 32°C for 48 h and periodically sampled and analyzed by high performance liquid chromatography (HPLC; as described below) to assess their comparative abilities with respect to producing 2-phenylethanol and 2-phenylacetate. Note that 2-phenylethanol and phenylacetate, which are endogenously produced from phenylpyruvate via ARO10 and either a native alcohol dehydrogenase (i.e., ADH1-6, and others putative dehydrogenases) or phenylacetaldehyde dehydrogenase (i.e., ALD1-6), respectively, were collectively used as surrogates to indicate enhanced flux through the phenylalanine biosynthesis pathway due to the fact that phenylalanine is not naturally exported by yeast to the extracellular media [24]. Several key pathway and regulatory genes (specifically, *ARO3*, *ARO4*, *ARO7*, *ARO8, GCN4*, and *PHA2*) were sequenced in each of BY4741 (wild type control) and top evolved mutants. In all cases, both the entire coding regions as well as 500 bp upstream of the start codon were sequenced.

### Transcriptional analysis of phenylalanine over-producing mutants

Relative transcription levels of each of *ARO1*, *ARO2*, *ARO3*, *ARO4*, *ARO7*, *ARO8*, *ARO9*, and *PHA2* were quantified at mid-log phase in each of BY4741 and the evolved mutants 22A and 22A75D. Approximately 1.5 × 10^8^ cells of each of strain were collected by centrifugation at 17,000 x *g* for 1 min. The supernatant was discarded and RNA was extracted from the cell pellet using the YeaStar RNA Extraction Kit (Zymo Research, Irvine, CA). cDNA was synthesized using the SuperScript VILO cDNA Synthesis Kit (Life Technologies) whereas RT-qPCR was performed using SYBR Green (Life Technologies) based quantitative PCR according to manufacturer’s protocols. Custom oligonucleotide primers for RT-qPCR experiments, including those for the reference housekeeping gene 26S [48], were designed and synthesized, the sequences of which are provided in Additional file 1: Table S2. RT-qPCR was performed on an Applied Biosystems StepOne Real-Time PCR (Applied Biosystems) using a 60°C annealing temperature. Data analysis was performed using StepOne software with relative transcriptional levels calculated via the 2^-ΔΔCt^ method [49].

### Investigating native induction and activity of FDC1

A seed culture of *S. cerevisiae* BY4741 were prepared in 5 mL YPD broth at 32°C while shaking at 250 rpm overnight. The same seed (1 mL) was then used to inoculate a series of sealed shake flasks (250 mL) each containing 50 ml SD media. Cultures were grown until reaching an OD_600_ of ~0.6, at which point either *trans*-cinnamate, ferulate, *p*-coumarate, or phenylalanine were added to a final concentration of 0.2 mM. All cultures were incubated for an additional 12 h after which an equal number of cells (~7.4x10^7^) were collected by centrifugation at 2,800 x *g* for 4 min. The cell pellet was lysed using Zymolyase (Zymo Research) before the sample was then centrifuged at 11,000 x *g* for 2 min to separate and collect the supernatant. Each sample was then assayed for its ability to catalyze the decarboxylation of *trans-*cinnamate to styrene by adding 5 μL of crude cell lysate to a 1 mL solution of 50 mM Tris–HCl buffer (pH 7.5) initially containing 250 mM *trans-*cinnamate. All samples were incubated at room temperature with the subsequent accumulation of styrene in the reaction mixture then followed at 247 nm for a total of 5 min at 20 s intervals using a UV/Vis spectrophotometer. A molar extinction coefficient of 10,000 M^−1^ cm^−1^ [50] and a 1 cm path length were used to establish activity in terms of mU mg^−1^ total protein. Total protein content in crude lysates was determined by Bradford Assay using bovine serum albumin (BSA) as an external standard.

### Cloning of PAL2 from A. thaliana

The *PAL2* encoding gene from *A. thaliana* was derived from a cDNA library plasmid containing the specific loci of interest (*AT3G53260*) and obtained from the Arabidopsis Biological Research Center (ABRC; Ohio State University, Columbus, OH). *PAL2* was PCR amplified using Phusion DNA Polymerase (Finnzymes, Espoo, Finland) and custom oligonucleotide primers (Additional file 1: Table S1). Using Gateway Cloning Technology [19], amplified linear DNA fragments flanked with *att*B sequences were purified using the Zyppy Clean and Concentrator kit (Zymo Research). The BP reaction between the DNA fragment and pDONR221 (Life Technologies) was performed using Gateway BP Clonase II Enzyme Mix (Life Technologies) following manufacturer’s protocols. Transformations were performed using chemically competent NEB10β with transformants being selected by plating overnight on LB solid agar containing 34 mg/L kanamycin. The resultant donor plasmid, pDONR-PAL, was mixed with the desired destination plasmid, p425GPD, using the Gateway LR Clonase II Enzyme Mix (Life Technologies), with transformation and selection as previously performed. As listed in Table 2, this approach resulted in construction of p425GPDPAL, a high copy (2 μ) plasmid for the constitutive expression of *PAL2* in *S. cerevisiae*.

### Assaying the extracellular transport potential of trans-cinnamate

*S. cerevisiae* BY4741 and the *FDC1* deletion mutant BY4741*ΔFDC1* were each transformed with plasmid p425GPDPAL, resulting in strains BY4741-PAL and BY4741*ΔFDC1*-PAL, respectively. All yeast transformations were performed by lithium acetate method [51]. Both strains were then cultured in SD-Leu media. Upon reaching OD_600_ of ~0.6, 200 mg/L phenylalanine was then added to each culture, after which the extracellular accumulation of each of *trans*-cinnamate, styrene, and 2-phenylethanol were then periodically monitored via HPLC for a total period of 24 h.

### Chromosomal disruption of ARO10 and integration of ARO4^K229L^

Targeted chromosomal disruption of *ARO10* in strain 22A75D was performed via homologous recombination. Gene disruption cassettes were generated via PCR to contain 40 base pairs of homology on both sides of the targeted integration site (i.e., *ARO10*), in addition to the KanMX selectable marker (as obtained from pFA6-KanMX4). Following transformation, colonies were selected on YPD solid agar plates containing 200 mg/L G418. Clones successfully carrying the *ARO10* disruption cassette were further confirmed by colony PCR. This resulted in the strain 22A75D10. In addition, a copy of the feedback resistant mutant *ARO4*^*K229L*^, whose expression was driven by the native *ARO4* promoter, was likewise integrated into 22A75D at the *ARO10* locus, thereby also and simultaneously resulting in the chromosomal disruption of *ARO10*. In this case, however, the gene disruption cassette was first constructed in the *E. coli* expression vector pACYCDuet-1. The ARO4 point mutation (K229L) was generated via overlap extension using primers as listed in Additional file 1: Table S1. The resultant fragment containing *ARO4*^*K229L*^ was inserted between the *BamHI* and *EcoRI* sites of pACYCDuet-1, before the desired mutation was then confirmed by sequencing. Subsequently, the KanMX selectable marker with its promoter was PCR amplified from pFA6-KanMX4 before then being inserted downstream of *ARO4*^*K229L*^ to generate the plasmid pACYC-ARO4^K229L^-KanMX. The entire cassette was then PCR amplified using primers whose overhangs each contained 40 base pairs of homology to *ARO10*. The resultant fragment was then transformed into 22A74D, followed by selection on YPD solid agar plates containing 200 mg/L G418. Successful clones were further confirmed by colony PCR, resulting in strain 22A75D104.

### Styrene production from glucose by S. cerevisiae

*S. cerevisiae* BY4741, 22A75D, 22A75D10, and 22A75D104 were each individually transformed with the plasmid p425GPDPAL. The resultant strains (BY4741-PAL, 22A75D-PAL, 22A75D10-PAL, and 22A75D104-PAL, respectively) were then each grown in 5 mL SD-Leu broth for 12 h at 32°C while shaking at 250 rpm to prepare seed cultures. Each seed (1 mL) was then used to inoculate 50 mL SD-Leu media in a 250 ml shake flask sealed with a glass cap. A closed system with large headspace was used to avoid volatile product (i.e., styrene) losses while also precluding the exhaustion of oxygen. Culturing continued for 48 h with periodic sampling performed to determine cell growth, residual substrate levels, and metabolite production.

### HPLC analysis

Substrate and metabolite concentrations were determined via HPLC analysis. Samples were prepared by first removing 1 mL of culture from shake flasks and pelleting the cells at 11,000 x *g* for 2 min. The supernatant (0.75 mL) was then transferred to a glass HPLC vial which was then sealed with a Teflon-lined screw cap. Separation and analysis was performed using a Hewlett Packard 1100 series HPLC system (Palo Alto, CA) equipped with an auto sampler, diode array (UV/Vis) detector, and reverse-phase Hypersil Gold SBC18 column (4.6 mm x 150 mm; Thermo Fisher, USA), according to previously described methods [10]. The eluent was monitored at 215 nm for phenylalanine, 2-phenylethanol, and 2-phenylacetate, and at 258 nm for *trans*-cinnamate and styrene. Using the same HPLC system, glucose analysis was performed using an RID detector and an anion exchange column (Aminex HPX-87H; BioRAD, Hercules, CA). The column was eluted with 0.005 M H_2_SO_4_ at a constant flow rate of 0.8 ml/min.

## Additional file

Addtional file 1:
**Further details of relevance to this study.**
