# Supplementary material for: Rational and combinatorial approaches to engineering styrene production by Saccharomyces cerevisiae
Source: Microb Cell Fact. 2014 Aug 21;13:123. doi: 10.1186/s12934-014-0123-2 (PMC4145238; doi:10.1186/s12934-014-0123-2)
Supplement: Addtional file 1: — Further details of relevance to this study. [file 12934_2014_123_MOESM1_ESM.docx]

**Supplementary Material**

| **Table S1.** Oligonucleotide primers designed for and used in the present study. | | |  |
| --- | --- | --- | --- |
| **Primers (5' to 3')** | |  |  |
| F_ARO10KanMX | TTATTTACAAGATAACAAAGAAACTCCCTTAAGCATGGCCGTACGCTGCAGGTCGAC | |  |
| R_ARO10KanMX | TGGTAGCAGTGTTTTATAATTGCGCCCACAAGTTTCTATATCGATGAATTCGAGCTCG | |  |
| F_ARO4_BamHI | ATAGGATCCATATTGACACTCTTTCATTGGGC | |  |
| R_ARO4_EcoRI | ATTGAATTCCTATTTCTTGTTAACTTCTCTTCTTTGTCT | |  |
| F_ARO4_OLE | TTCATGGGTGTTACTTTGCATGGTGTTGCTGCTATCACCACTA | |  |
| R_ARO4_OLE | AGCAGCAACACCATGCAAAGTAACACCCATGAAATGGTGAGAA | |  |
| F_PAL2_attB1 | GGGGACAAGTTTGTACAAAAAAGCAGGCTAAAAAAATGTCTCAAATCGAAGCAATGTTG | |  |
| R_PAL2_attB2 | GGGGACCACTTTGTACAAGAAAGCTGGGTTTAGCAAATCGGAATCGGAGCT | |  |
| F_ARO1_qPCR | GCTACAGTTTCTTCTCAGTACG | |  |
| R_ARO1_qPCR | CGACGTACAATTTAGAGATTGG | |  |
| F_ARO2_qPCR | CCTCACGACTACTCCGACAT | |  |
| R_ARO2_qPCR | CGTTTCTCTAGCAGAAGCTCT | |  |
| F_ARO3_qPCR | GCTGGAAAGGGTTGATTAAC | |  |
| R_ARO3_qPCR | CCAACATCTCACCAGCAAT | |  |
| F_ARO4_qPCR | CGAATCTCAACTGCACAGA | |  |
| R_ARO4_qPCR | GCTTGACAAGCATCCACAG | |  |
| F_ARO7_qPCR | GGTTCTGTTGCCACTAGAGAT | |  |
| R_ARO7_qPCR | GCTTTGTGTATAGCGGGATAT | |  |
| F_ARO8_qPCR | CGACTTCCTAATTGTGGAAGAT | |  |
| R_ARO8_qPCR | GCTTTGGAGAACTTTGTGC | |  |
| F_ARO9_qPCR | GCTACAGGAGCAAAAGTCATC | |  |
| R_ARO9_qPCR | CCAGTTGACCAATTATCGAG | |  |
| F_PHA2_qPCR | GGATTGTTCTTCCACATCTG | |  |
| R_PHA2_qPCR | GCTGTTTCACTAGCAATGG | |  |
| F_26S_qPCR | CCTATGATTTGAGTATCTCAGC | |  |
| R_26S_qPCR | CGTAATTGGAATCGTTGACTAT | |  |

| **Table S2.** Sequencing primers designed for and used in the present study. | |  |
| --- | --- | --- |
| F1_ARO3_seq | ATGTTCATTAAAAACGATCACG |  |
| F2_ARO3_seq | GCACAGAGAATTAGCATCCG |  |
| F3_ARO3_seq | GCTGTAGAACCTGTTGTCACTT |  |
| R1_ARO3_seq | CGTTCTTAAATCCAATAGGGAA |  |
| R2_ARO3_seq | TTTTTTCAAGGCCTTTCTTCTG |  |
| R3_ARO3_seq | CCTTTGATTCTCCAGTCTTCC |  |
| F1_ARO4_seq | ATGAGTGAATCTCCAATGTTCG |  |
| F2_ARO4_seq | GCCAGAACCACCGAATCTCAAC |  |
| R1_ARO4_seq | GGCCAATTCTCTGTGCAGTT |  |
| R2_ARO4_seq | TTTCTTGTTAACTTCTCTTCTTTGT |  |
| F1_ARO7_seq | ATGGATTTCACAAAACCAGAAA |  |
| F2_ARO7_seq | CATTAATTTCGAAAAGAGATGGT |  |
| R1_ARO7_seq | CCGAAGTTATTCTTATCATCACC |  |
| R2_ARO7_seq | CTCTTCCAACCTTCTTAGCAAG |  |
| F1_GCN4_seq | ATGTCCGAATATCAGCCAAGT |  |
| F2_GCN4_seq | GCAATTGAATCCACTGAAGAAG |  |
| R1_GCN4_seq | CCAGATTGGATGGTACCAGA |  |
| R2_GCN4_seq | GCGTTCGCCAACTAATTTCT |  |
| F1_PHA2_seq | CGTACTACATCATCTGCGACA |  |
| R1_PHA2_seq | GCAGCTGTTTCACTAGCAAT |  |
| F2_PHA2_seq | GCAGGTCACCTTTATAAGATTG |  |
| R2_PHA2_seq | GCCAGGTTTAAGCATATAAAAGTG |  |

**Table S3**. Common known regulators of *ARO1*, *ARO2*, *ARO3*, and *ARO8* expression.

| Regulator | Association Type  (if known) | Description | Reference |
| --- | --- | --- | --- |
| ACE2 | Negative | Transcription factor required for septum destruction after cytokinesis; phosphorylation by Cbk1p blocks nuclear exit during M/G1 transition, causing localization to daughter cell nuclei, and also increases Ace2p activity; phosphorylation by Cdc28p and Pho85p prevents nuclear import during cell cycle phases other than cytokinesis; part of RAM network that regulates cellular polarity and morphogenesis; ACE2 has a paralog, SWI5, that arose from the whole genome duplication | [1] |
| BAS1 |  | Myb-related transcription factor; involved in regulating basal and induced expression of genes of the purine and histidine biosynthesis pathways; also involved in regulation of meiotic recombination at specific genes | [2] |
| GCN4 | Positive | bZIP transcriptional activator of amino acid biosynthetic genes; activator responds to amino acid starvation; expression is tightly regulated at both the transcriptional and translational levels | [3]  [4]  [5] |
| LEU3 | Negative | Zinc-knuckle transcription factor, repressor and activator; regulates genes involved in branched chain amino acid biosynthesis and ammonia assimilation; acts as a repressor in leucine-replete conditions and as an activator in the presence of alpha-isopropylmalate, an intermediate in leucine biosynthesis that accumulates during leucine starvation | [6]  [2] |
| RAD3 |  | 5' to 3' DNA helicase; involved in nucleotide excision repair and transcription; subunit of RNA polII initiation factor TFIIH and of Nucleotide Excision Repair Factor 3 (NEF3); homolog of human XPD protein; mutant has aneuploidy tolerance; protein abundance increases in response to DNA replication stress | [2] |
| SOK2 | Positive | Nuclear protein that negatively regulates pseudohyphal differentiation; plays a regulatory role in the cyclic AMP (cAMP)-dependent protein kinase (PKA) signal transduction pathway; relocalizes to the cytosol in response to hypoxia; SOK2 has a paralog, PHD1, that arose from the whole genome duplication | [7]  [8] |
| SSL1 |  | Subunit of the core form of RNA polymerase transcription factor TFIIH; has both protein kinase and DNA-dependent ATPase/helicase activities; essential for transcription and nucleotide excision repair; interacts with Tfb4p | [2] |
| SWI3 |  | Subunit of the SWI/SNF chromatin remodeling complex; SWI/SNF regulates transcription by remodeling chromosomes; contains SANT domain that is required for SWI/SNF assembly; is essential for displacement of histone H2A-H2B dimers during ATP-dependent remodeling; required for transcription of many genes, including ADH1, ADH2, GAL1, HO, INO1 and SUC2; relocates to the cytosol under hypoxic conditions | [2] |
| TAF1 |  | TFIID subunit, involved in RNA pol II transcription initiation; possesses in vitro histone acetyltransferase activity but its role in vivo appears to be minor; involved in promoter binding and G1/S progression; relocalizes to the cytosol in response to hypoxia | [2] |
| VPS72 |  | Htz1p-binding component of the SWR1 complex; exchanges histone variant H2AZ (Htz1p) for chromatin-bound histone H2A; may function as a lock that prevents removal of H2AZ from nucleosomes; required for vacuolar protein sorting | [2] |
| YRM1 | Not applicable | Zinc finger transcription factor involved in multidrug resistance; Zn(2)-Cys(6) zinc finger transcription factor; activates genes involved in multidrug resistance; paralog of Yrr1p, acting on an overlapping set of target genes | [9] |

**References**

1. Di Talia S, Wang H, Skotheim JM, Rosebrock AP, Futcher B, Cross FR: **Daughter-specific transcription factors regulate cell size control in budding yeast.** *PLoS Biol* 2009, **7:**e1000221.

2. Venters BJ, Wachi S, Mavrich TN, Andersen BE, Jena P, Sinnamon AJ, Jain P, Rolleri NS, Jiang C, Hemeryck-Walsh C, Pugh BF: **A comprehensive genomic binding map of gene and chromatin regulatory proteins in *Saccharomyces*.** *Mol Cell* 2011, **41:**480-492.

3. Moxley JF, Jewett MC, Antoniewicz MR, Villas-Boas SG, Alper H, Wheeler RT, Tong L, Hinnebusch AG, Ideker T, Nielsen J, Stephanopoulos G: **Linking high-resolution metabolic flux phenotypes and transcriptional regulation in yeast modulated by the global regulator Gcn4p.** *Proc Natl Acad Sci U S A* 2009, **106:**6477-6482.

4. Natarajan K, Meyer MR, Jackson BM, Slade D, Roberts C, Hinnebusch AG, Marton MJ: **Transcriptional profiling shows that Gcn4p is a master regulator of gene expression during amino acid starvation in yeast.** *Mol Cell Biol* 2001, **21:**4347-4368.

5. Uluisik I, Kaya A, Unlu ES, Avsar K, Karakaya HC, Yalcin T, Koc A: **Genome-wide identification of genes that play a role in boron stress response in yeast.** *Genomics* 2011, **97:**106-111.

6. Vuralhan Z, Luttik MA, Tai SL, Boer VM, Morais MA, Schipper D, Almering MJ, Kotter P, Dickinson JR, Daran JM, Pronk JT: **Physiological characterization of the ARO10-dependent, broad-substrate-specificity 2-oxo acid decarboxylase activity of *Saccharomyces cerevisiae.*** *Appl Environ Microbiol* 2005, **71:**3276-3284.

7. Rossouw D, Jacobson D, Bauer FF: **Transcriptional regulation and the diversification of metabolism in wine yeast strains.** *Genetics* 2012, **190:**251-261.

8. Vachova L, Devaux F, Kucerova H, Ricicova M, Jacq C, Palkova Z: **Sok2p transcription factor is involved in adaptive program relevant for long term survival of Saccharomyces cerevisiae colonies.** *J Biol Chem* 2004, **279:**37973-37981.

9. Lucau-Danila A, Delaveau T, Lelandais G, Devaux F, Jacq C: **Competitive promoter occupancy by two yeast paralogous transcription factors controlling the multidrug resistance phenomenon.** *J Biol Chem* 2003, **278:**52641-52650.
